# An Impact Load History Reconstruction Method for Composite Structures Based on FBG Sensing Data and the GCV Principle

**DOI:** 10.3390/s26092601

**Published:** 2026-04-23

**Authors:** Jie Zeng, Jihong Xu, Yuntao Xu, Xin Zhao, Shiao Wang, Yanwei Zhou, Yuxun Wang

**Affiliations:** 1 College of Aerospace Engineering, Nanjing University of Aeronautics and Astronautics, Nanjing 210016, China; 2 AVIC Chengdu Caic Electronics Co., Ltd., Chengdu 610000, China

**Keywords:** composite structures, fiber Bragg grating, impact load history reconstruction, modified Tikhonov regularization, generalized cross-validation

## Abstract

**Highlights:**

**What are the main findings?**

- We propose an impact load history reconstruction method for composite structures based on GCV and improved regularization, which enables effective waveform reconstruction from sparse strain data acquired from low-rate quasi-distributed FBG sensors.
- We establish a multi-dimensional quantitative indicator system (including RMSE, MAE, PRE, CC, and PLD) to comprehensively evaluate the reconstruction performance in terms of the waveform profile restoration and transient detail characterization.

**What are the implications of the main findings?**

- The proposed GCV-based method overcomes the limitations of conventional Tikhonov regularization by adaptively optimizing the regularization parameter, achieving superior anti-interference capability, waveform consistency, and transient peak identification accuracy in complex noisy environments.
- The established indicator system provides a scientific basis for quantitatively comparing different reconstruction methods regarding their identification accuracy, waveform consistency, and peak tracking capability, thus supporting the health monitoring and digital twin modeling of aircraft composite structures.

**Abstract:**

Accurately and promptly acquiring the load history characteristics of impact events on composite aircraft structures is crucial for identifying impact-induced damage and developing high-fidelity digital twin models. To address this need, we propose a method for reconstructing the impact load history on composite structures, leveraging Generalized Cross-Validation (GCV) and a Fiber Bragg Grating (FBG) pattern. An equivalent expansion technique based on discretized time-domain sparse strain sampling is developed to mitigate the local distortion of impact response signals, a common issue arising from the low sampling rates of quasi-distributed FBG. By incorporating Tikhonov regularization, the ill-posed nature of the impact frequency response matrix is effectively managed. Furthermore, an adaptive optimization method based on the GCV criterion is introduced to overcome the limitations of manually selecting regularization parameters and the associated constraints on noise suppression. The results show that the proposed GCV-based reconstruction method achieves an average peak relative error of 11.4% and an average root mean square error of 0.36 N for the reconstructed impact load, demonstrating that the proposed method synergistically enhances both the reconstruction of the overall impact load waveform profile and the precise characterization of transient details, even with low-rate sampling. This provides robust technical support for health monitoring and condition-based maintenance of composite structures.

## 1. Introduction

Composite materials offer advantages such as high specific strength and stiffness, excellent fatigue resistance, and design flexibility, and are extensively used in critical aircraft components including wings, fuselages, control surfaces, and engine nacelles [1,2]. However, during service, these composite structures are inevitably subjected to impact loads from events such as projectile impacts, bird strikes, hail, runway debris, and tool drops, which can induce internal damage such as delamination, fiber breakage, and matrix cracking. If not detected promptly, this damage can propagate under subsequent service loads, leading to performance degradation and potentially catastrophic failure [3]. Consequently, research into impact load monitoring and history reconstruction is crucial for assessing the impact severity, developing high-fidelity digital twin models, and enabling predictive maintenance and remaining useful life prediction for aircraft [4,5].

Traditional piezoelectric sensor arrays, with their excellent high-frequency response, are highly capable of sensing transient impact events within specific local areas of composite structures [6,7]. By constructing high-frequency piezoelectric sensor arrays, the high-frequency wave response caused by impacts can be captured in detail, enabling impact localization and history reconstruction. However, this approach imposes significant demands on the data processing capabilities, real-time transmission bandwidth, and storage capacity of onboard monitoring systems [8].

Crucially, the dynamic strain response and its distribution characteristics in composite structures subjected to transient impacts are closely related to the features of the impact load history [9]. Therefore, integrating strain sensing arrays with the structure provides correlative data that are essential for reconstructing the impact load history [10]. Conventional resistive strain sensing, however, requires extensive wiring for signal transmission, which not only complicates the monitoring system but can also alter the natural frequency of the structure under test [11,12]. In contrast, quasi-distributed FBG offers unique advantages, including light weight, a small diameter, multi-parameter synchronous sensing, ease of distributed networking, and straightforward integration with composites. This makes it a promising integrated and lightweight monitoring solution for efficiently capturing impact response characteristics in primary aircraft load-bearing structures [13]. Several researchers have applied this technology for the identification of strain field characteristics in composite structures and the evaluation of fatigue performance under dynamic service environments [14,15], indicating that Fiber Bragg Grating sensors can be extended to the field of load characteristic identification for composite materials.

Beyond acquiring transient impact response data, achieving high-quality reconstruction of the impact load on composite structures is a primary research focus. Recent research has explored model-based methods for load history reconstruction. These methods establish an accurate forward physical model (“impact load–strain response”) and frame the reconstruction as an inverse optimization problem. For instance, Zhou et al. [16] proposed a method for reconstructing the time-domain history of impact forces on composite structures by combining Lp-norm minimization with a forward structural dynamics model, creating a physically meaningful reconstruction framework. Kalhori et al. [17] developed an algorithm for identifying the impact forces on composite laminates based on the cosine similarity matching of frequency response characteristics. By establishing a mapping between the high-frequency response of piezoelectric sensors and the transfer function, they isolated the impact load using deconvolution techniques. Other researchers have employed machine learning algorithms for direct learning from extensive datasets. For example, Seno et al. [18] proposed a data-driven stochastic Kriging method that uses feature space interpolation and uncertainty quantification to enhance the robustness of impact force reconstruction. Tian et al. [19] introduced a load feature identification algorithm for composite structures combining particle swarm optimization and back-propagation neural networks. This method directly learns the complex nonlinear mapping between the load and response from data through global parameter optimization and nonlinear modeling.

It is important to note that model-based reconstruction methods heavily rely on the accuracy of the mechanical model representing the structure under test. However, real aircraft composite structures are subject to uncertainties such as manufacturing process variability, non-ideal boundary conditions, and in-service thermo-mechanical coupling. These factors make it challenging for established mechanical models to accurately represent the dynamic response characteristics of the actual structure [20,21]. Such model errors are particularly amplified under low sampling rates, leading to severe ill-posedness in the inverse load reconstruction process [22]. While data-driven methods avoid complex modeling, they require constructing a substantial training dataset covering various aircraft configurations, impact locations, and energy levels, which results in high implementation costs and difficulty in covering all possible in-service impact scenarios [23]. Encountering an unknown impact pattern outside the training set or structural performance degradation over time can significantly reduce the model generalization, leading to distorted reconstruction results, especially when the sampling rates are limited [24].

Given the stringent constraints on the data processing capacity and real-time transmission bandwidth in practical onboard monitoring systems, developing impact load reconstruction methods that operate on low-rate discrete sensing patterns while maintaining strong generalization capability is a critical challenge [25,26,27]. Although the sampling frequencies of FBGs are considerably lower than those of piezoelectric sensors, an FBG can directly measure the strain response and distribution caused by transient impacts. This facilitates the creation of quasi-distributed sensor networks which are suitable for monitoring large-scale aircraft load-bearing components, resulting in a simpler and more efficient system with significant monitoring advantages [28].

To address these challenges, we propose a method for monitoring and reconstructing the impact load history on composite panels, based on low-rate quasi-distributed FBG sensing data combined with the Generalized Cross-Validation (GCV) criterion. The key contributions are as follows. (1) An equivalent expansion technique for impact response signals is developed, based on discretizing the dynamic response and sparse time-domain strain sampling. This technique aims to overcome the loss of detailed impact response information caused by the inherently low sampling rates of quasi-distributed FBG sensors. (2) A Tikhonov regularization framework is constructed to transform the load identification problem into a stable inverse solution, addressing the numerical instability and reconstructed waveform oscillation arising from the ill-posed nature of the impact load frequency response matrix derived from sparse time-domain strain data. (3) An adaptive optimization method for the Tikhonov regularization parameter, based on the GCV criterion, is innovatively proposed to balance the inherent trade-off between solution smoothness and detail fidelity during regularization. (4) Building upon this, a multi-dimensional quantitative indicator system is established to comprehensively evaluate the effectiveness of impact load history reconstruction. This system assesses the performance in terms of the overall waveform profile restoration and the accurate characterization of transient details, providing a scientific basis for quantitatively comparing different reconstruction methods regarding identification accuracy, waveform consistency, and peak tracking capability.

## 2. Methodology for Impact Load History Reconstruction

### 2.1. Ill-Posedness Analysis of the Frequency Response Matrix

In load identification problems, modeling errors in the mechanical description of the structure and the presence of sensor noise can render the frequency response matrix ill-conditioned, which makes the identified results highly sensitive to minor perturbations, often leading to severe oscillations or even divergence in the reconstructed load vector—a phenomenon characterized as significant ill-posedness [29]. To mitigate such issues, a quantitative assessment of the ill-posedness inherent in the inverse solution process is necessary [30].

To address the local distortion of reconstructed impact response signals and the ill-posedness caused by the low sampling rates of quasi-distributed FBG sensing data, we propose an impact response signal expansion method based on dynamic discretization modeling and sparse strain sampling. By constructing a discrete time-domain “strain–load” mapping model and applying singular value decomposition and generalized inverse theory, the expression for the impact load solution vector F is obtained as follows [31]:

(1) $$F=H^{-1}Y=\sum_{i=1}^{Q_{0}}\frac{u_{i}^{T}Y}{\sigma_{i}}v_{i}$$

where *H* is the initial frequency response matrix, *Y* is the strain response vector acquired by the FBG sensor array, and *Q_0_* is the number of non-zero singular values of *H*. Equation (1) shows that, when *H* is ill-posed, if certain singular values *σ_i_* approach zero, the corresponding coefficients |*u_i_^T^Y*|/*σ_i_* become highly amplified. In particular, if the measured strain response *Y* contains even minor noise, these small singular values can amplify it significantly, causing the identified result to deviate substantially from the true load.

Mathematically, the well-posedness of such an inverse problem can be evaluated using the Picard condition. This condition requires that the coefficients |*u_i_^T^Y*| decay faster than the singular values *σ_i_* [32]; that is,

(2) $$\frac{\left|u_{i}^{T}Y\right|}{\sigma_{i}}\to 0.$$

When the smallest singular value of the initial frequency response matrix *H* approaches zero, the Picard condition is often violated. Consequently, the coefficients corresponding to these tiny singular values are disproportionately amplified during the solution process, transforming minor noise in the measured response data into severe oscillations. Thus, a stable solution cannot be obtained using conventional least-squares methods.

### 2.2. Tikhonov Regularization for Load Reconstruction

Tikhonov regularization introduces a penalty term to filter noise, balancing data fidelity with the plausibility of the solution. This effectively mitigates the adverse effects of the measurement noise on the uniqueness and stability of the inverse solution. Therefore, a Tikhonov regularization method is proposed to address the ill-posedness inherent in the inverse reconstruction of impact loads. The corresponding functional is expressed as [33]

(3) $$J={\left\|\mathrm{HF}-Y\right\|}_{2}^{2}+\eta {\left\|F\right\|}_{2}^{2},$$

where *η* is a filtering adjustment operator. By setting the first derivative of the Tikhonov objective function *J* with respect to the solution vector *F* to zero, the Tikhonov-regularized solution for the impact load, *F_η_*, can be derived from the discrete impact response signals as [34]

(4) $$F_{\eta }=\sum_{i=1}^{Q_{0}}\frac{\sigma_{i}^{2}}{\eta +\sigma_{i}^{2}}\frac{u_{i}^{T}Y}{\sigma_{i}}v_{i},$$

where *Q*_0_ is the number of singular values of *H* greater than zero, *σ_i_* are the non-negative singular values, *u_i_^T^Y*/*σ_i_* are the combination coefficients, and *v_i_* are the elements of the right singular vectors.

To satisfy the Picard condition and obtain a convergent approximate solution, specific regularization functions are employed to attenuate the high-frequency noise. The core idea is to pre-multiply the initial frequency response matrix *H* by a regularization filtering operator [35], yielding an optimized frequency response matrix *H^#^*. Based on Equation (1), combining *H^#^* with the measured FBG sensing data response signals allows one to solve for the regularized impact load vector:

(5) $$H^{\#}=\sum_{i=1}^{Q_{0}}g_{\lambda }^{\gamma }(\sigma_{i})\frac{u_{i}^{T}}{\sigma_{i}}v_{i}.$$

In Equation (5), *H^#^* is the optimized frequency response matrix, and *g^λ^_γ_*(*σ_i_*) is the regularization operator, which essentially serves a filtering function. Its specific form is defined as [36]

(6) $$g_{\lambda }^{\gamma }(\sigma_{i})=\frac{\sigma_{i}^{\gamma }}{\lambda +\sigma_{i}^{\gamma }}\;\;,\;\;\gamma \geq 0.$$

In Equation (6), *λ* is the regularization parameter, which satisfies *λ* > 0; and *γ* is the filtering parameter matrix, which satisfies *γ* ≥ 0. The operator *g_λ_^γ^*(*σ_i_*) is continuous and depends on *γ*; as *γ* approaches 0, *g_λ_^γ^*(*σ_i_*) approaches 1. When *γ* = 2, Equation (6) represents the influence matrix for the standard Tikhonov regularization method.

It is important to note that the regularization parameter *λ* directly governs the filtering effect of the operator *g_λ_^γ^*(*σ_i_*) on the singular values *σ_i_*. Therefore, determining the optimal regularization parameter *λ_opt_* is a critical step for improving the Tikhonov method.

Once *λ_opt_* is found, the improved Tikhonov-regularized solution vector for the impact load, *F_λopt_*, can be computed from the discrete impact response signals [37]:

(7) $$F_{\lambda_{\mathrm{opt}}}=\sum_{i=1}^{Q_{0}}\frac{\sigma_{i}^{\gamma }}{\lambda_{\mathrm{opt}}+\sigma_{i}^{\gamma }}\frac{u_{i}^{T}Y}{\sigma_{i}}v_{i}.$$

An excessively large regularization parameter, while effectively suppressing the measurement noise, can also discard valuable information inherent in the system model, thereby compromising the accuracy of impact load identification. To address this, we further propose using the L-curve method, as well as the Generalized Cross-Validation method, to determine the optimal regularization parameter *λ_opt_*, aiming to adaptively balance the trade-off between the fidelity and accuracy of the reconstructed load history.

### 2.3. Improved Tikhonov Regularization with L-Curve Parameter Optimization

The core idea of the improved Tikhonov regularization method based on L-curve parameter optimization for the frequency response matrix (hereafter referred to as the L-curve method) is to characterize, on a logarithmic scale, the relationship between the L_2_ norm of the regularized load solution vector *P*_1_(*λ*) (i.e., the L_2_ norm ||*LF_λ_*||_2_) and the L_2_ norm of the residual *P*_2_(λ) (i.e., the L_2_ norm ||*HF_λ_*−*Y*||_2_). Each point on this curve has coordinates (log||*LF_λ_*||_2_, log||*HF_λ_*−*Y*||_2_). By identifying the corner of the L-curve (i.e., the point of maximum curvature), the optimal value of the improved Tikhonov regularization parameter λ_opt_ is determined, which is then used to obtain the optimized frequency response matrix H^#^.

(8) $$\left\{\begin{array}{c} P_{1}(\lambda )=\log\left|\right|{\mathrm{LF}}_{\lambda }|{|}_{2}\\ P_{2}(\lambda )=\log\left|\right|HF_{\lambda }-Y|{|}_{2} \end{array}\right.$$

At the corner of the L-curve, both the solution error and the perturbation error are relatively small, which helps to minimize the impact load reconstruction error. Based on the coordinates of this inflection point, combined with Equation (7) in Section 2.1 and Equation (8) in this section, the corresponding optimal regularization parameter *λ_opt_* can be determined.

To accurately identify the corner (point of maximum curvature), the curvature *K*(*λ*) of the L-curve is calculated as follows:

(9) $$K(\lambda )=\frac{\left|{P_{1}}^{\prime }{P_{2}}^{\prime \prime }-{P_{2}}^{\prime }{P_{1}}^{\prime \prime }\right|}{{\left(P_{1}^{2}+P_{2}^{2}\right)}^{3/2}},$$

where *P*_1_′ and *P*_2_′ represent the first derivatives of *P*_1_*(λ*) and *P*_2_*(λ*), respectively; and *P*_1_″ and *P*_2_″ represent their second derivatives.

### 2.4. Improved Tikhonov Regularization with GCV Parameter Optimization

When the L-curve is too smooth to allow for clear identification of a distinct corner, it can lead to bias in selecting the optimal regularization parameter *λ_opt_*. To address this limitation, we also propose an improved Tikhonov regularization method based on Generalized Cross-Validation parameter optimization for the frequency response matrix (hereafter referred to as the GCV method) as an alternative approach for determining the optimal parameter. The Generalized Cross-Validation objective function is defined as

(10) $$G(\lambda )=\frac{(1/m)||(I-C(\lambda )Y|{|}^{2}}{{\left\{\left(1/m)tr[(I-C(\lambda ))\right]\right\}}^{2}},$$

where *C*(*λ*) = *HH^#^* represents the product of the initial frequency response matrix *H* and the optimized frequency response matrix *H^#^*, m is the number of rows in *H*, and *I* is the identity matrix.

**Figure 1:**
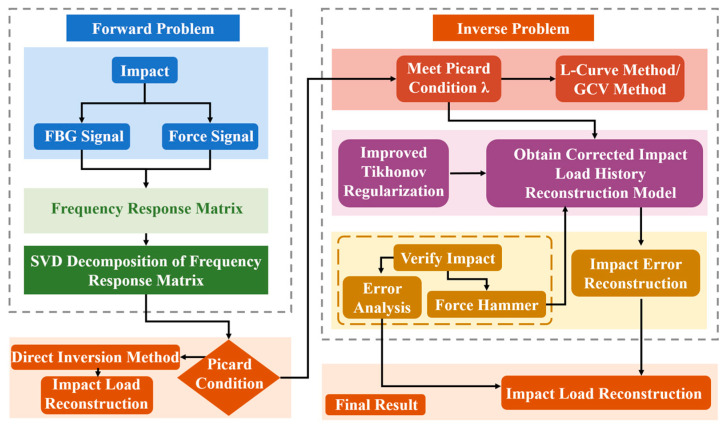
Framework of improved Tikhonov regularization method for impact history reconstruction based on Generalized Cross-Validation criterion.

Compared to the L-curve method, the advantage of the GCV method lies in its criterion function, which consistently yields a distinct minimum. This makes the adaptive identification process for the optimal regularization parameter more intuitive and deterministic. The framework for the improved Tikhonov regularization method for impact history reconstruction, utilizing GCV-based parameter optimization for the frequency response matrix, is illustrated in Figure 1.

## 3. Simulation Validation

### 3.1. Finite Element Modeling of Impact Response

**Figure 2:**
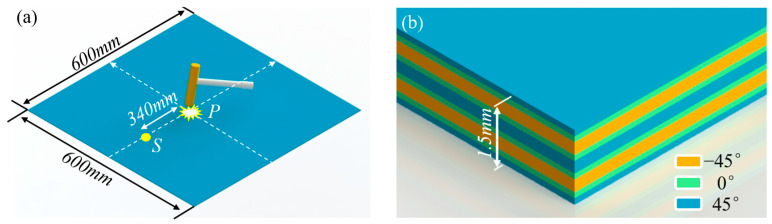
Finite element model of composite laminate impact response: (**a**) low-velocity impact response simulation model; (**b**) composite laminate stacking sequence.

A dynamic simulation model for the low-velocity impact response of a composite laminate was established, with the specific geometric configuration shown in Figure 2a. The composite laminate had planar dimensions of 600 mm × 600 mm and a thickness of 1.5 mm. The laminate was clamped on all four sides as the boundary conditions. In Figure 2a, point *P* denotes the impact location and point *S* denotes the strain extraction location.

In the numerical simulation, a rigid impact hammer was used to apply the impact load. The geometric and material parameters of the hammer were as follows: the steel hammerhead was hemispherical with a radius of 2 mm, a density of 7.8 g/cm^3^, an elastic modulus of 210 GPa, and a Poisson’s ratio of 0.3.

The objectives of the numerical simulation were as follows: first, to reveal the influence of the number of sampling points on the ill-conditioning of the frequency response matrix and to clarify the limitations of the direct inversion method in load reconstruction; second, to validate the effectiveness of the GCV criterion in the adaptive selection of regularization parameters, thereby providing a theoretical basis for determining the regularization parameters in subsequent experiments; and third, to compare the impact load identification accuracy of different reconstruction methods and to preliminarily evaluate the feasibility of the proposed approach.

The composite laminate was fabricated using prepreg with a single-layer thickness of 0.125 mm. A total of 12 layers were laid, resulting in a total thickness of 1.5 mm. The stacking sequence from bottom to top was designed as a symmetric layup, specifically [45°/0°/−45°/−45°/0°/45°]_S_, as shown in Figure 2b. The basic mechanical property parameters of the material used for each layer are detailed in Table 1.

### 3.2. Ill-Posedness Analysis of the Initial Frequency Response Matrix Using the Direct Inversion Method

**Figure 3:**
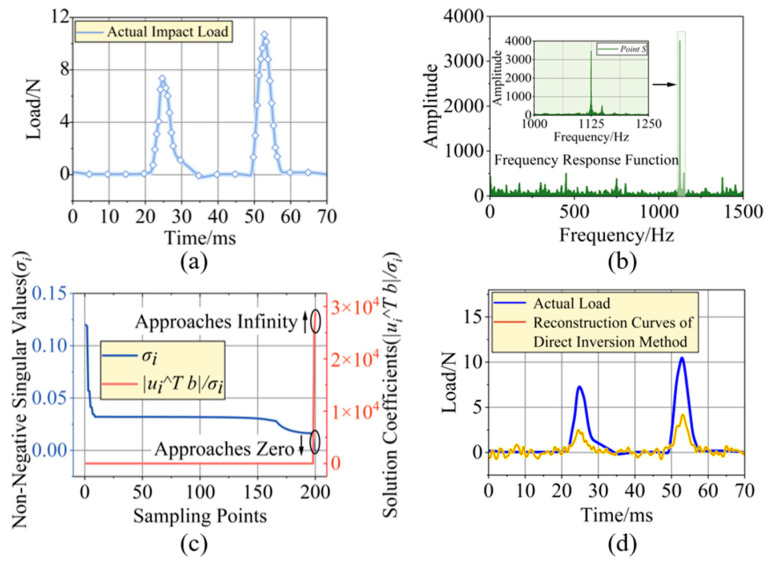
Simulation-based evaluation of the load reconstruction method using direct inversion: (**a**) actual impact load history, (**b**) the frequency response relationship between the impact point and the strain extraction point, (**c**) Picard well-posedness analysis of the direct inversion method (the horizontal axis represents the number of sampling points; under a fixed sampling frequency, an increase in the number of sampling points corresponds to a lengthening of the sampling time window), and (**d**) the impact load history reconstruction result using the direct inversion method.

The impact load history applied in the numerical simulation is shown in Figure 3a. The impact load response corresponding to point *P* (impact location) and the strain signal corresponding to point *S* (strain extraction location) in Figure 2a were substituted into Equation (1) to obtain the system frequency response matrix. To reduce the impact of random noise on frequency response matrix identification, in the simulation, impact loads with the same parameters but different added random noises were applied three times. The frequency response matrices obtained from the three independent runs were then averaged to obtain the characteristics of the initial frequency response matrix *H*, as shown in Figure 3b. The magnitude of the elements of the initial frequency response matrix reached a peak at a frequency of 1125 Hz.

This frequency response matrix is only applicable to the currently specified impact location (Point *P*) and strain response measurement location (Point *S*) and cannot be directly used for impact identification at other locations. If the impact location changes, the stress wave propagation path and modal response characteristics will change accordingly, necessitating the reconstruction of a corresponding frequency response matrix for the new location. In practical applications, the multi-location impact identification problem can be addressed by pre-constructing transfer functions covering all possible impact locations, which is also a key direction for future research.

The results of the well-posedness analysis for the direct inversion method are shown in Figure 3c. As the number of sampling points increased (which corresponded to a longer sampling time window under a fixed sampling frequency), more low-frequency components could be captured. Consequently, the singular values *σ_i_* of the constructed initial frequency response matrix tended toward zero, causing the correlation coefficients |*u_i_^T^Y*|/*σ_i_* to tend towards infinity. This violated the Picard condition, indicating that the inversion process was ill-posed. The impact load history reconstruction results obtained using the direct inversion method also confirmed this ill-posedness. The solution vector obtained by direct inversion exhibited divergent oscillations, leading to significant reconstruction errors, as shown in Figure 3d.

To address this issue, further adoption of improved regularization methods was necessary to obtain a stable approximate solution vector.

### 3.3. Calculation of the Optimal Regularization Parameter λopt for the Improved Regularization Method for Initial Frequency Response Matrix Optimization

**(1) Calculation of the optimal regularization parameter *λ_opt_* using the improved regularization method based on the L-curve reconstruction method.**

**Figure 4:**
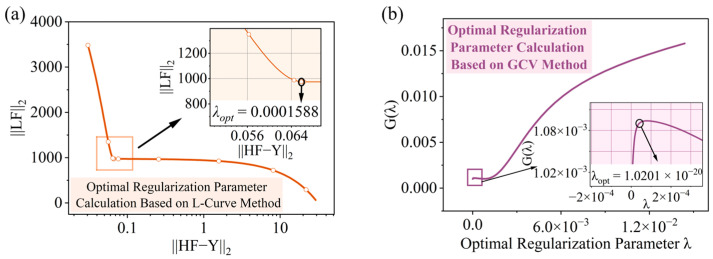
Optimization results for the optimal regularization parameter *λ_opt_* using the improved Tikhonov regularization method: (**a**) the relationship curve between the L_2_ norm of the regularized solution vector *P*_1_*(λ*) and the L_2_ norm of the residual *P*_2_*(λ*) based on the L-curve reconstruction method; (**b**) the Generalized Cross-Validation function curve G(λ) based on the GCV reconstruction method.

The L-curve reconstruction method determines the optimal regularization parameter ***λ**_opt_* by locating the point of maximum curvature, i.e., the corner of the L-curve. According to the method described in Section 2.3, the calculated ***λ**_opt_* was 0.0001588, and the result is shown in Figure 4a. In this figure, the abscissa represents the L_2_ norm of the residual ||*HF_λ_*-*Y*||_2_, and the ordinate represents the L_2_ norm of the regularized solution vector||*LF_λ_*||_2_.

**(2) Calculation of the optimal regularization parameter *λ_opt_* using the improved regularization method based on the GCV reconstruction method.**

According to the method described in Section 2.4, the GCV reconstruction method identified the optimal regularization parameter ***λ_opt_*** = 1.0201 × 10^−20^ by solving for the minimum point of the Generalized Cross-Validation function value G(λ), as shown in Figure 4b. The abscissa represents the regularization parameter λ (on a logarithmic scale), and the ordinate represents the Generalized Cross-Validation function value G(λ). Compared to the L-curve reconstruction method, the optimal regularization parameter ***λ_opt_*** obtained by the GCV reconstruction method exhibited higher resolution, indicating that this method has better sensitivity in parameter selection. Simultaneously, the GCV reconstruction method demonstrated a faster numerical approximation speed, enabling a more efficient response to abrupt detail features in the impact load history and facilitating the determination of a more stable and adaptable optimal regularization parameter.

### 3.4. Simulation Validation of the Impact Load History Reconstruction Performance

**Figure 5:**
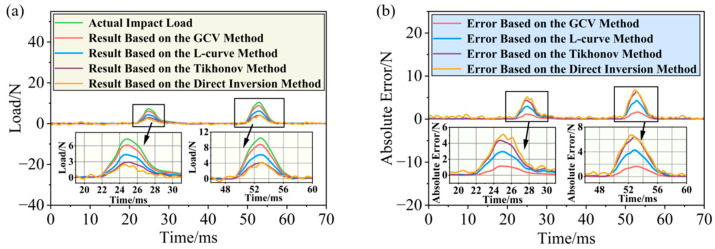
Performance evaluation of different impact load reconstruction methods based on numerical simulation: (**a**) impact load history reconstruction curves; (**b**) absolute errors of impact load history reconstruction.

Validation was performed using the direct inversion method, the Tikhonov regularization method, and the improved Tikhonov regularization methods (including the L-curve reconstruction method and the GCV reconstruction method). The reconstruction results corresponding to each method are shown in Figure 5a.

The simulation results indicated that the direct inversion method, which did not incorporate a regularization term, produced reconstruction results severely contaminated by noise. The reconstructed waveform exhibited pronounced oscillations and deviated substantially from the true load. Although the Tikhonov regularization method suppressed some high-frequency oscillations, it yielded reconstructed values in the peak region that were considerably lower than the true load. Moreover, the load solution vector obtained by the L-curve-based method also showed notable discrepancies from the overall profile of the actual impact load history. In contrast, the load solution vector reconstructed by the GCV-based method achieved a high level of agreement with the true impact load history, both in terms of the overall waveform profile and the detailed peak characteristics.

To further quantitatively evaluate the accuracy of the impact load history reconstruction using different methods, the root mean square error (RMSE) corresponding to the direct inversion method, the Tikhonov regularization reconstruction method, the L-curve reconstruction method, and the GCV reconstruction method was calculated. The RMSE values decreased progressively, at 1.801 N, 1.588 N, 1.068 N, and 0.413 N, respectively. Correspondingly, the relative errors in reconstructing the peak impact load also showed a decreasing trend, at 62.05%, 60.61%, 40.66%, and 15.89%, respectively.

These results demonstrate that the GCV reconstruction method, by adaptively selecting the optimal regularization parameter, overcame the limitation of the Tikhonov reconstruction method, where the regularization parameter heavily relied on subjective experience. It also avoided the issue encountered by the L-curve reconstruction method in identifying the point of maximum curvature in flat regions of the curve. Consequently, the GCV method significantly enhanced both the reconstruction of the overall profile and the fidelity of local detail features for transient impact load histories.

## 4. Experimental Validation and Discussion

### 4.1. Experimental System Setup

**Figure 6:**
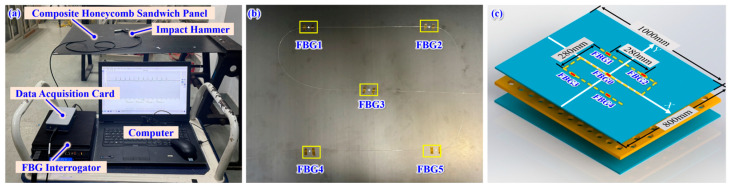
Impact load history monitoring and reconstruction system: (**a**) impact load monitoring and reconstruction system for composite honeycomb sandwich panel based on FBG, (**b**) physical layout of FBG, and (**c**) layout scheme of FBG.

To evaluate the performance of different impact load history reconstruction methods, an experimental system was established, as shown in Figure 6a. The system primarily consisted of the following components: a composite honeycomb sandwich panel, a clamped support frame, a PCB impact hammer, FBG, an NI data acquisition card, a Si255 multi-channel FBG interrogator (with a sampling frequency of 5 kHz), and a computer.

The composite honeycomb sandwich panel had dimensions of 1000 mm in length, 800 mm in width, and a thickness of 4.5 mm. It was fixed to the support frame using a single-edge clamped boundary condition.

A quasi-distributed FBG sensing network, consisting of FBG_0_, FBG_1_, FBG_2_, FBG_3_, and FBG_4_, was bonded onto the surface of the composite honeycomb sandwich panel to monitor the dynamic strain response of the panel under impact loads in real time.

A Cartesian coordinate system was established with the location of sensor FBG_0_ as the origin. The specific coordinate positions of the quasi-distributed FBG sensor array layout are shown in Figure 6c.

### 4.2. Experimental Procedure

**Figure 7:**
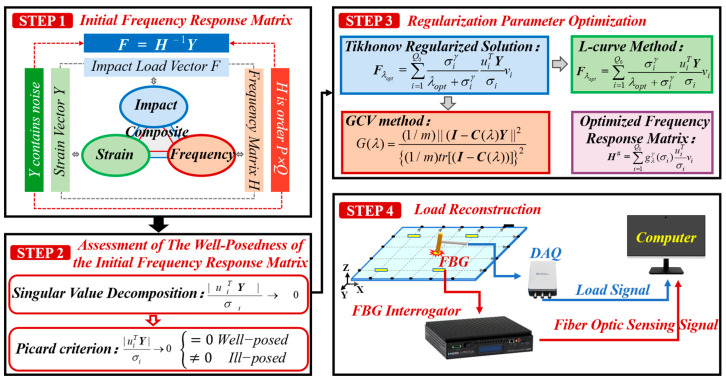
Experimental procedure for impact load monitoring and history reconstruction on a composite panel using the L-curve-based or GCV-based reconstruction method.

The experimental procedure for impact load monitoring and history reconstruction using FBG sensors, as shown in Figure 7, was as follows.

**STEP 1:** For each selected impact location, n transient impacts were pre-applied. Based on the excitation signal from the impact hammer and the response characteristics of the FBG sensors for each impact, the corresponding frequency response matrices were calculated. By averaging the elements of the frequency response matrices obtained from these *n* pre-tests, the initial frequency response matrix *H* corresponding to each impact location was constructed.

**STEP 2:** Singular value decomposition was performed on the initial frequency response matrix *H* to extract its singular value distribution and the left and right singular vectors. The Picard condition was then applied to assess the well-posedness of this initial frequency response matrix.

**STEP 3:** If the initial frequency response matrix was found to be ill-posed, a regularization operator was introduced. The optimal regularization parameter *λ_opt_* was determined using either the L-curve reconstruction method or the Generalized Cross-Validation reconstruction method. Subsequently, by incorporating *λ_opt_* into Equation (5), the optimized frequency response matrix *H^#^* was obtained.

**STEP 4:** Impact experiments were conducted on the composite honeycomb sandwich panel. The response signals from the FBG sensors and the actual load history data measured by the impact hammer were acquired synchronously. By substituting the strain response signals collected by the FBG sensors along with the optimized frequency response matrix *H^#^* into Equation (7), the impact load history was reconstructed.

### 4.3. Experimental Results of Impact Load History Reconstruction

**Figure 8:**
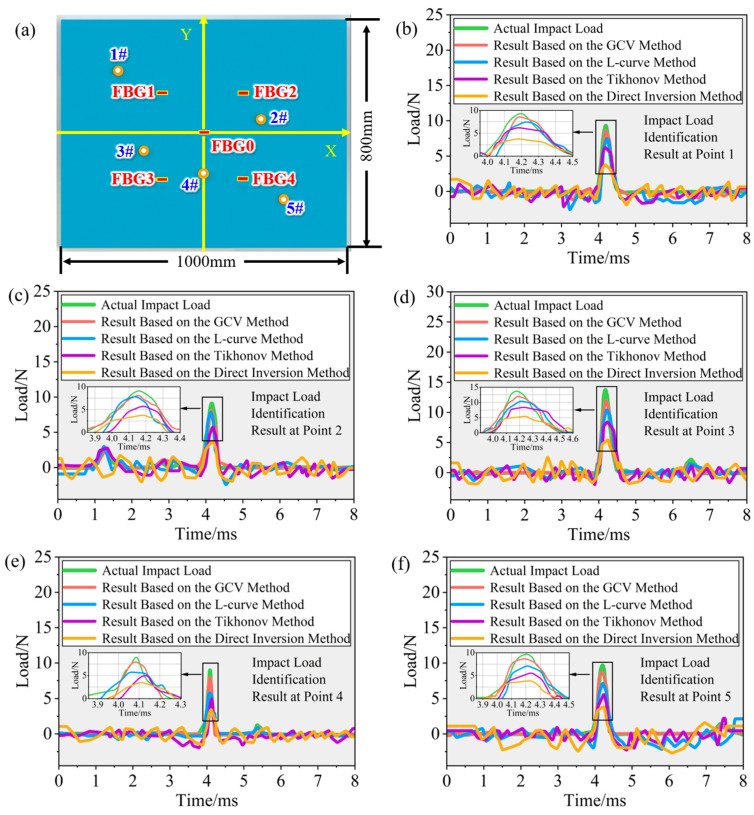
Impact load history reconstruction results: (**a**) distribution of different impact load locations, (**b**) load history reconstruction results for impact point 1#, (**c**) load history reconstruction results for impact point 2#, (**d**) load history reconstruction results for impact point 3#, (**e**) load history reconstruction results for impact point 4#, and (**f**) load history reconstruction results for impact point 5#.

Five impact verification points were randomly set on the surface of the composite honeycomb sandwich panel, as shown in Figure 8a. These impact locations covered both the central and edge regions of the panel to comprehensively evaluate the applicability of the relevant methods for load history reconstruction at different impact positions.

Taking impact point 1# as an example, the load history reconstruction results obtained using the direct inversion method, the Tikhonov regularization method, the L-curve-based method, and the GCV-based method are shown in Figure 8b. The reconstructed waveform obtained by the direct inversion method was severely contaminated by noise, exhibiting severe oscillations and deviating from the true load curve. The Tikhonov regularization method effectively suppressed high-frequency oscillations; however, the reconstructed amplitude at the impact peak was significantly lower than the true value, and the overall waveform exhibited distortion. The waveform identified by the L-curve-based method closely approximated the overall profile of the true load history, but there remained a noticeable phase shift and amplitude deviation near the impact peak. In contrast, the waveform identified by the GCV-based method showed good agreement with the actual impact load history in terms of waveform profile, peak timing, and amplitude characteristics, demonstrating superior waveform restoration and tracking capability.

Similarly, for impact points 2#, 3#, 4#, and 5#, the impact load history reconstruction results obtained using the four different methods exhibited consistent trends, as shown in Figure 8c–f, respectively. The GCV criterion enabled adaptive selection of the regularization parameter, avoiding the subjectivity of manual trial and error.

### 4.4. Evaluation of Impact Load History Reconstruction Performance

**(1)**  **Quantitative Evaluation of Impact Load History Reconstruction Accuracy**

**Figure 9:**
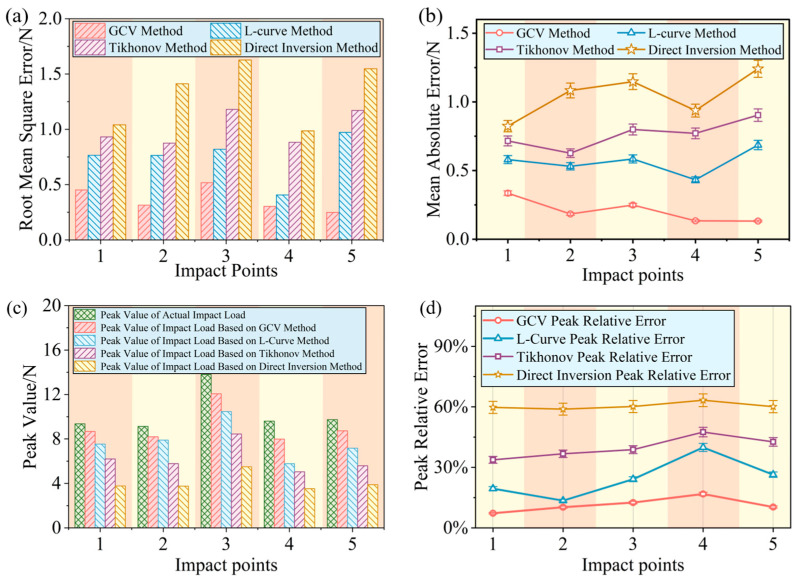
Comparison of experimental results for impact load reconstruction: (**a**) RMSE corresponding to different reconstruction methods, (**b**) absolute errors corresponding to different reconstruction methods, (**c**) reconstructed peak loads versus actual peak loads for different methods, and (**d**) peak identification relative errors for different reconstruction methods.

Using the root mean square error (RMSE), absolute error, and peak identification relative error as evaluation metrics, the reconstruction error curves for impact load histories obtained by using the four different methods were calculated, as shown in Figure 9.

The average RMSE of the load history reconstruction for each impact point using the direct inversion method was 1.32 N. The average RMSE for the Tikhonov regularization method was 1.01 N, and for the L-curve-based method, it was 0.74 N. In contrast, the average RMSE for the GCV-based method was significantly reduced to 0.36 N, indicating its superior reconstruction accuracy and stability.

The average absolute error of load history reconstruction for each impact point using the direct inversion method was 1.05 N. The average absolute error for the Tikhonov regularization method was 0.76 N, and for the L-curve-based method, it was 0.56 N. In contrast, the average absolute error for the GCV-based method was significantly reduced to 0.21 N.

The Peak Relative Error (PRE) for the reconstructed impact load peak is defined as

(11) $$\mathrm{PRE}=\frac{\left|\max(Fr)-\max(F)\right|}{\left|\max(F)\right|}\times 100\%,$$

where *max*(*Fr*) and *max*(*F*) represent the reconstructed peak load and the actual peak load, respectively.

Calculations based on Equation (11) show that the average PRE for each impact point using the direct inversion method was 60.4%. The average PRE for the Tikhonov regularization method was 39.8%, and for the L-curve-based method, it was 24.6%. In contrast, the average PRE for the GCV-based method was significantly reduced to 11.4%.

These results demonstrate that the GCV-based method possesses a significant accuracy advantage in impact load history reconstruction. This is primarily attributed to its ability to adaptively determine the regularization parameter, thereby achieving an optimal balance between fitting accuracy and noise immunity and avoiding the over-smoothing or over-fitting problems caused by overly conservative or aggressive selection of the regularization parameter. Compared with the direct inversion method without regularization, the peak error of the load reconstructed using the proposed method was effectively reduced, and the oscillation phenomenon was significantly suppressed.

**(2)**  **Waveform Consistency Evaluation of Impact Load Reconstruction**

**Figure 10:**
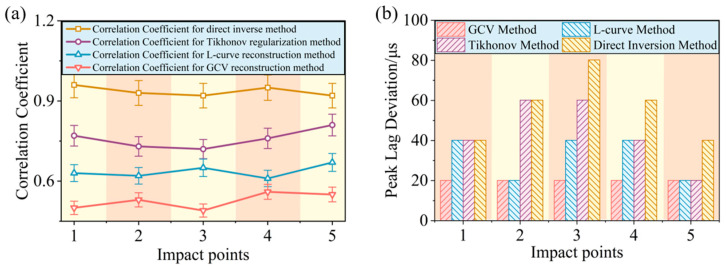
Evaluation of waveform consistency and peak tracking capability for impact load reconstruction: (**a**) waveform correlation coefficients between the load identification curves obtained by the four reconstruction methods and the actual load history, and (**b**) peak lag deviation indices corresponding to the four reconstruction methods.

To further evaluate the waveform consistency between the impact load curves obtained by the four reconstruction methods and the actual impact load history, a quantitative correlation analysis was performed. The mathematical expression for the correlation coefficient CC is

(12) $$\mathrm{CC}=\frac{\sum_{i=1}^{n}\left[Fr_{i}-E(Fr)\right]\;\left[F_{i}-E(F)\right]}{\left\|Fr-E(Fr)\right\|\;\left\|F-E(F)\right\|},$$

where Fr is the reconstructed load, F is the actual load, and E(Fr) and E(F) are the mean values of the reconstructed load and the actual load, respectively. The distribution curves of the waveform correlation coefficients between the reconstructed impact load curves and the actual impact load history for the four methods at different impact points are shown in Figure 10a.

The average waveform correlation coefficient for the load histories reconstructed by the direct inversion method at each impact point was only 0.526, making it difficult to capture the true load variation history. The average waveform correlation coefficient for the Tikhonov regularization method was 0.636 and, for the L-curve-based method, it was 0.758, indicating significant distortion and deviation between the load reconstruction waveforms obtained by using these two methods and the true load history. In contrast, the average waveform correlation coefficient for the GCV-based method reached 0.936, demonstrating good waveform consistency and temporal correlation between the reconstructed impact load curve and the actual load history. Compared with the L-curve criterion, the GCV method exhibited better stability under low signal-to-noise ratio conditions.

**(3)**  **Peak Tracking Capability Evaluation of Impact Load Reconstruction Waveforms**

To assess the peak tracking capability of the four reconstruction methods, the Peak Lag Deviation (PLD) index was introduced for analysis. Due to certain differences in the steepness of the curves in the peak region of the loads reconstructed by the four methods, deviations in peak time identification may occur. Therefore, the time corresponding to the maximum peak of the reconstructed load waveform is defined as *T_r_*(1), with two adjacent times, *T_r_*(2) through *T_r_*(5), taken on its left and right sides. Similarly, the time corresponding to the maximum peak of the actual load waveform is defined as *T*(1), with two adjacent times, *T*(2) through *T*(5), taken on its left and right sides. The PLD index based on these five impact time instants was calculated using the following formula:

(13) $$\mathrm{PLD}=\frac{1}{5}\sum_{i=1}^{5}\left|T_{r}(i)-T(i)\right|$$

where *T_r_*(*i*) is the i-th time instant of the reconstructed load waveform, and *T*(*i*) is the i-th time instant of the actual load waveform.

The average PLD for the direct inversion method was 52.13 μs. The average PLD for the Tikhonov regularization method was 44.11 μs and, for the L-curve-based method, it was 32.08 μs. In contrast, the average PLD for the GCV-based method was reduced to 20.05 μs. These results indicate that the GCV-based method exhibits the best peak tracking performance for load identification, primarily due to its faster numerical approximation speed, which enables a more efficient response to abrupt features in the impact load history.

## 5. Conclusions

To meet the requirements of health monitoring and digital twin construction for aircraft composite structures, we proposed an impact load history reconstruction method based on GCV and an improved regularization approach. By employing low-sampling-rate quasi-distributed FBG sensing technology, effective reconstruction of transient impact load waveforms from sparse strain signals was achieved. This method also provides a theoretical reference for solving inverse problems of flexible structure deformation based on tomographic principles. The main contributions of this paper are as follows:

(1) An equivalent expansion method for impact response signals based on time-domain sparse strain sampling was proposed. Combined with Tikhonov regularization, this method effectively addresses the numerical instability and waveform oscillation issues caused by the ill-conditioned frequency response matrix under low sampling rates.

(2) An adaptive optimization method for regularization parameters based on the GCV criterion was established. This approach overcomes the limitations of traditional methods, which rely on empirical parameter selection and insufficient noise suppression capability, achieving an adaptive balance between reconstruction fit and accuracy.

(3) A multi-dimensional quantitative evaluation system was constructed, encompassing the root mean square error, peak relative error, waveform correlation coefficient, and peak lag deviation. This provides a scientific basis for assessing the reconstruction accuracy, waveform consistency, and peak tracking performance of impact load reconstruction.

## Figures and Tables

**Table 1:** Basic material properties of the laminate layers.

*E*_1_ (GPa)	*E*_2_ (GPa)	*N*_*u*12_	*G*_12_ (GPa)	*G*_13_ (GPa)	*G*_23_ (GPa)	*ρ* (g/cm^3^)
131	9.0	0.3	5.4	5.4	4.5	1.58

## Data Availability

The original contributions presented in this study are included in the article. Further inquiries can be directed to the corresponding author.
